# Supplementary material for: Improvement of cryo-EM maps by simultaneous local and non-local deep learning
Source: Nat Commun. 2023 Jun 3;14:3217. doi: 10.1038/s41467-023-39031-1 (PMC10239474; doi:10.1038/s41467-023-39031-1)
Supplement: Supplementary file 1 — Supplementary Information [file 41467_2023_39031_MOESM1_ESM.pdf]

Supplementary Information

Improvement of cryo-EM maps by simultaneous local and non-local deep learning

Jiahua He, Tao Li, and Sheng-You Huang*

School of Physics and Key Laboratory of Molecular Biophysics of MOE, Huazhong University of Science and Technology, Wuhan, Hubei 430074, China.

*Email: huangsy@hust.edu.cn; Phone: +86-27-87543881; Fax: +86-027-87556576

Supplementary Table 1: Map interpretability of *de novo* model building. The results of phenix.map_to_model are evaluated on a test set of 682 chains. The results of MAINMAST are evaluated on a test set of 385 chains. The numbers in bold fonts indicate the best performances for the corresponding metrics.

Method	phenix.map_to_model		MAINMAST	
	Coverage (%)	Sequence match (%)	Coverage (%)	Sequence match (%)
deposited	64.2	31.9	73.9	15.2
DeepEMhancer	58.1	34.6	72.1	14.6
phenix.auto_sharpen	64.3	32.9	74.0	15.4
**EMReady**	**79.7**	**50.4**	**85.6**	**33.8**

Supplementary Table 2: Summary of the ablation results. The ablation experiments of EMReady are carried out on the test set of 110 deposited primary cryo-EM maps and the test set of 25 pairs of half-maps. The numbers in bold fonts indicate the best performances for the corresponding metrics on each test set. Asterisks denote statistically significant differences determined by the two-sided Wilcoxon signed-rank test in comparison with the baseline model, i.e. '*' denotes $p<0.01$, '**' denotes $p<0.001$, and '***' denotes $p<0.0001$. The exact p values for the test set of primary maps and the test set of half-maps can be found in Supplementary Data 7 and 8, respectively.

Test set	EMReady model	FSC-0.5 (Å)	Q-score	CC_box	CC_mask	CC_peaks
primary maps	box size=24	3.82***	0.521***	0.854	**0.803*****	0.750
	box size=32	3.64***	0.533***	0.852	0.799***	0.749*
	UNet++	3.61***	0.532***	0.852	0.795	0.752
	w/o SSIM	3.63***	0.523***	0.838***	0.788***	0.747**
	**baseline**	**3.57**	**0.542**	**0.855**	0.798	**0.753**
half-maps	box size=24	4.32***	0.467***	0.871	**0.794**	0.750
	box size=32	4.27***	0.471***	0.870	0.790	0.755
	UNet++	4.24***	0.468***	0.863*	0.787	0.748
	w/o SSIM	4.29**	0.463***	0.851***	0.781***	0.748*
	**baseline**	**4.07**	**0.491**	**0.873**	**0.794**	**0.760**

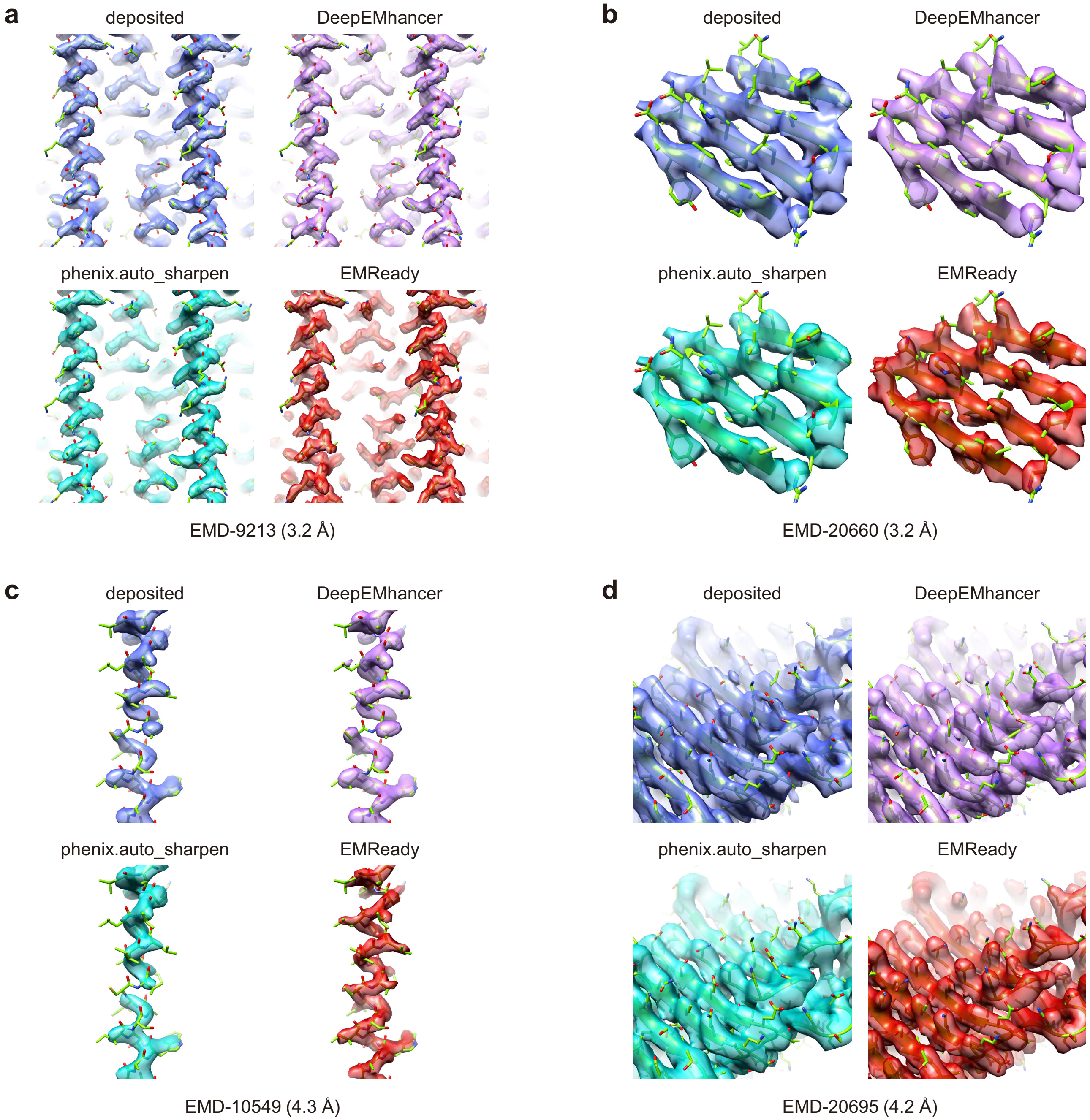

Supplementary Fig. 1: Examples of the EMReady-processed maps compared with the deposited maps and the maps processed by other methods. **a** α-helices in EMD-9213 (reported resolution: 3.2 Å). **b** β-sheet in EMD-20660 (reported resolution: 3.2 Å). **c** An α-helix in EMD-10549 (reported resolution: 4.3 Å). **d** β-sheet in EMD-20695 (reported resolution: 4.2 Å). The deposited PDB structures are colored in green. Contours are drawn to enclose equal volumes for each case.

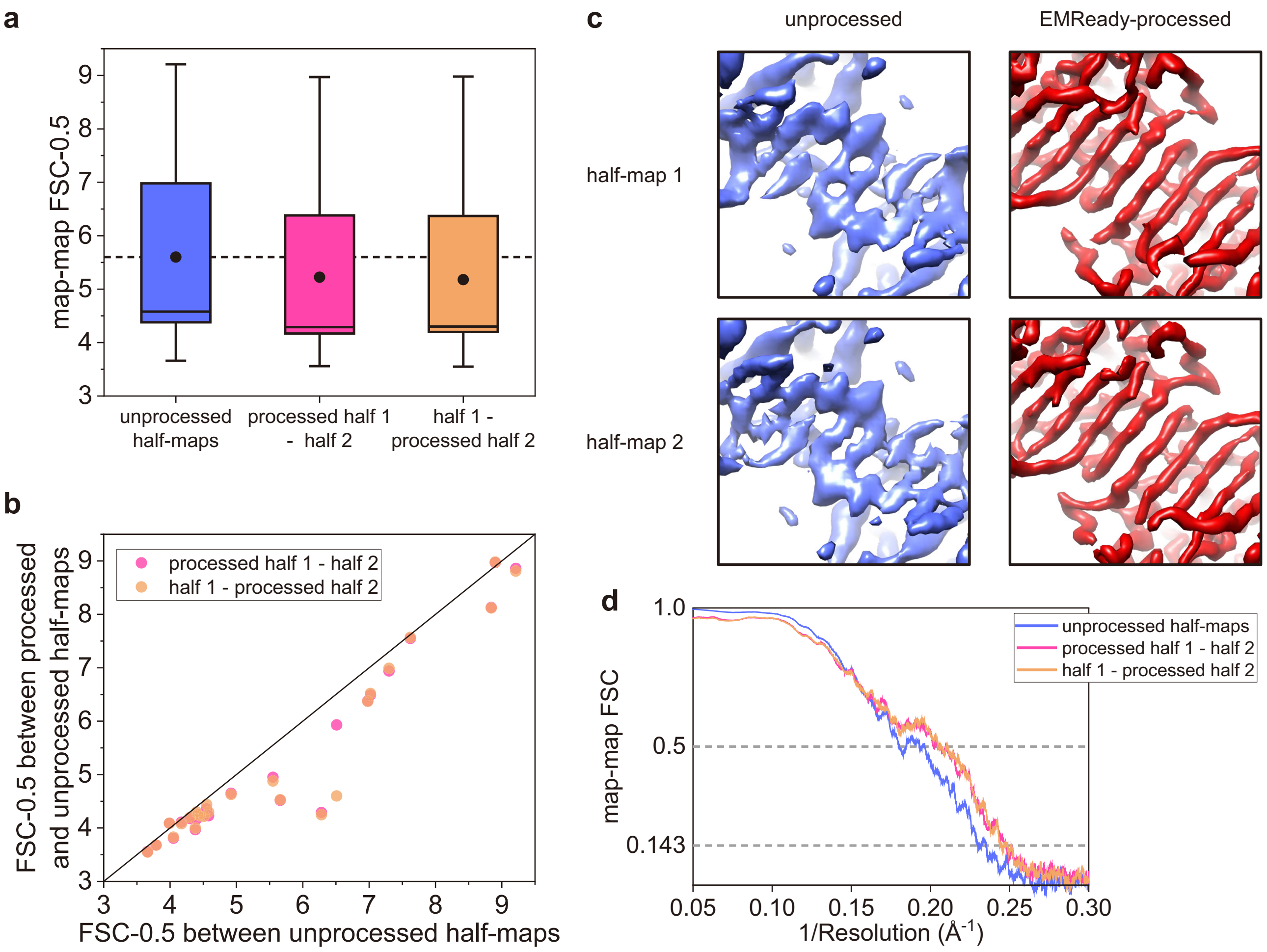

Supplementary Fig. 2: Applying EMReady to one half-map and evaluating against the other unprocessed half-map on the test set of 25 pairs of half-maps. a Box-whisker plot of unmasked map-map FSC-0.5 between two unprocessed half-maps and between one EMReady-processed half-map and the other unprocessed half-map (n=25 individual test cases). The center line is the median, the circle is the mean, lower and upper hinges represent the first and third quartile, the whiskers stretch to 1.5 times the interquartile range from the corresponding hinge. The dashed line stands for the average FSC-0.5 between unprocessed half-maps. **b** Comparison of the unmasked FSC-0.5 between two unprocessed half-maps and FSC-0.5 between one EMReady-processed half-map and the other unprocessed half-map on each test case. **c** An example of the unprocessed and EMReady-processed half-maps of EMD-0071. **d** The unmasked map-map FSC curves between two unprocessed half-maps and between one EMReady-processed half-map and the other unprocessed half-map of EMD-0071. Source data are provided as a Source Data file.

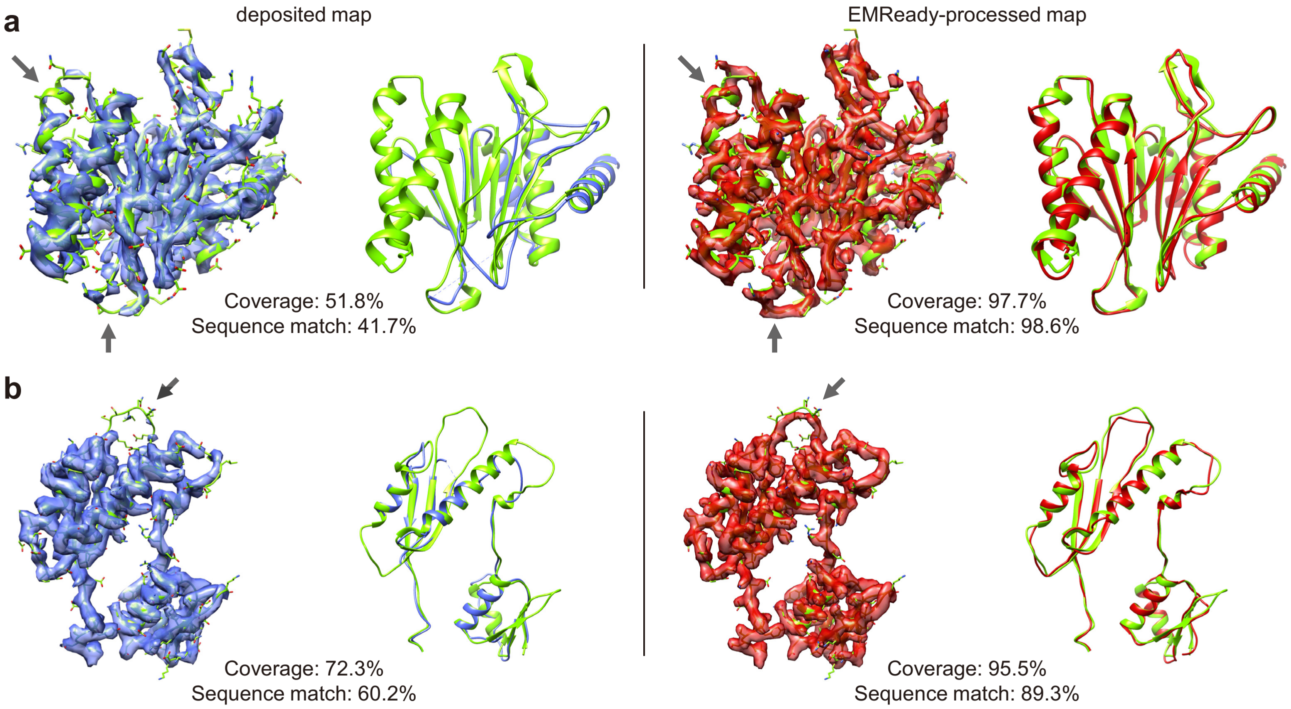

Supplementary Fig. 3: Examples of the models built by phenix.map_to_model on the deposited map and EMReady-processed map. **a** 5LZP_L on EMD-4128. **b** 6RWX_h on EMD-10045. The density volumes and corresponding models built by phenix.map_to_model for the deposited maps are colored in blue and those for EMReady-processed maps are in red. The reference PDB model is colored in green. Contours are drawn to enclose equal volumes for each case. Arrows indicate regions of density signals that are enhanced by EMReady.

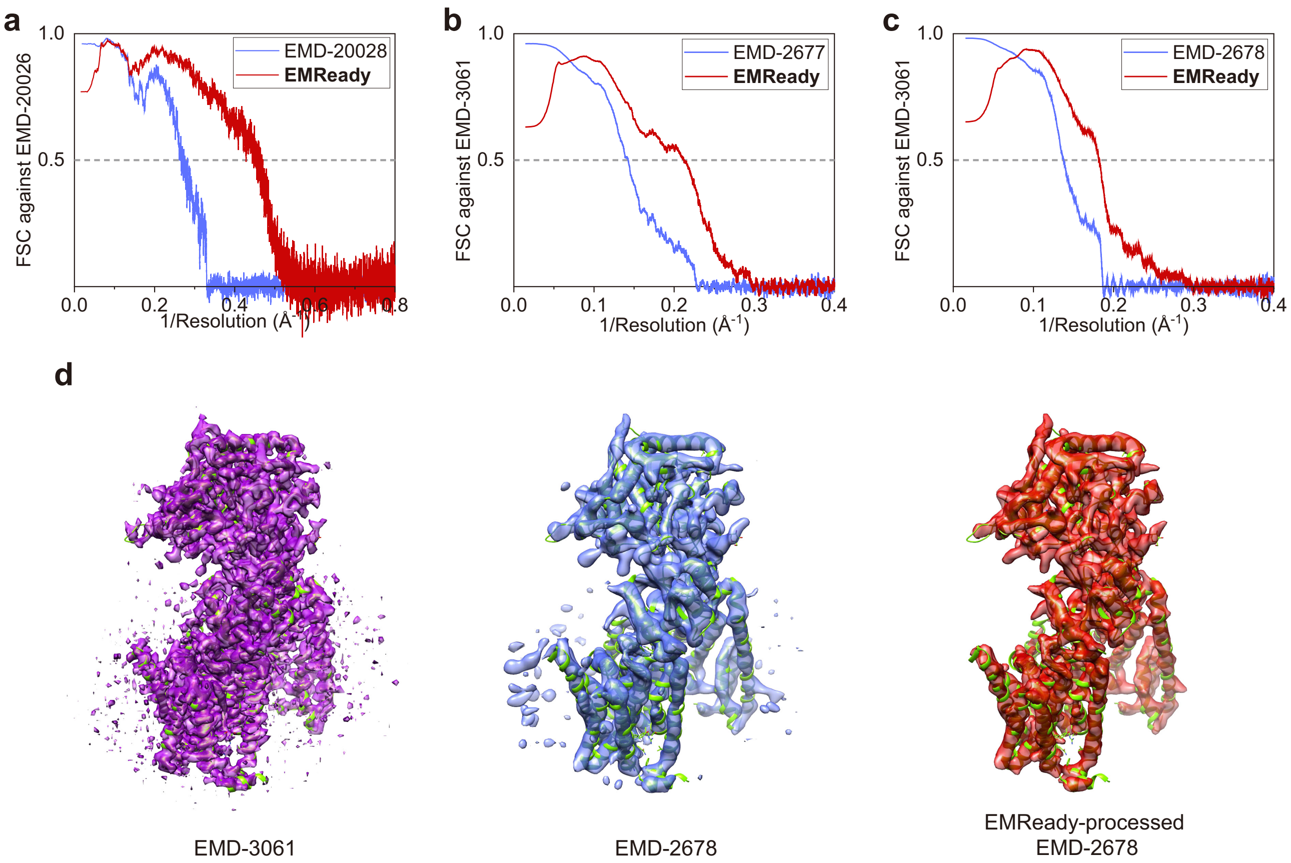

Supplementary Fig. 4: Applying EMReady to lower-resolution maps and evaluating against higher-resolution cryo-EM maps. a–c Comparison the unmasked map-map Fourier Shell Correlation (FSC) versus the inverse resolution between the deposited primary map and the EMReady-processed map for 3.1-Å EMD-20028 against 1.8-Å EMD-20026 (**a**), 4.5-Å EMD-2677 against 3.4-Å EMD-3061 (**b**), and 5.4-Å EMD-2678 against 3.4-Å EMD-3061 (**c**), respectively. **d** Comparing the density volumes for EMD-3061 (purple), EMD-2678 (blue), and the EMReady-processed map of EMD-2678 (red). Contours are drawn to enclose equal volumes. The associated PDB structure of EMD-3061 (PDB ID: 5A63) is colored in green. Source data are provided as a Source Data file.

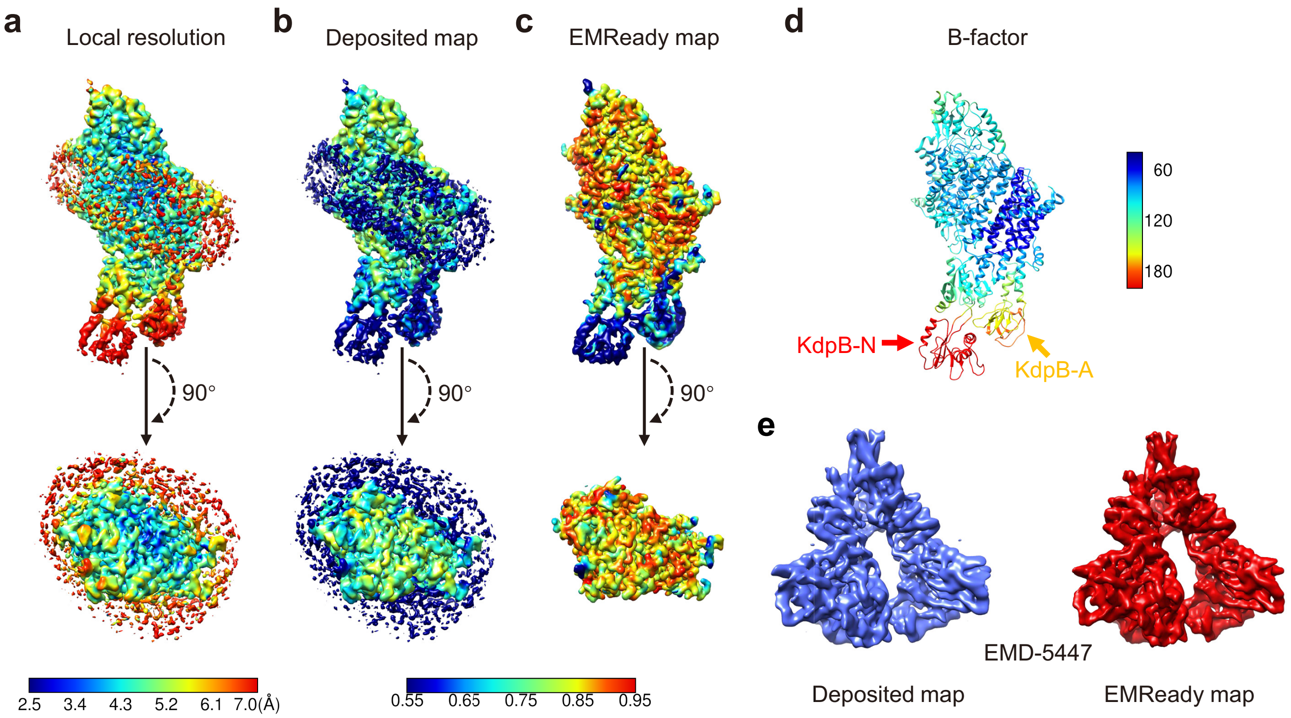

Supplementary Fig. 5: Impact of local map quality on EMReady. a–d Analysis of EMD-0257 at 3.7 Å resolution: deposited map colored by local resolution (**a**), deposited map colored by its local correlation with the simulated map (**b**) EMRead-processed map colored by its local correlation with the simulated map (**c**), and associated PDB structure (PDB ID: 6HRA) colored by B-factor (**d**). **e** Comparison of the deposited map (blue, *left panel*) and the EMReady-processed map (red, *right panel*) for EMD-5447 at 6.0 Å resolution. Contours are drawn to enclose equal volumes.

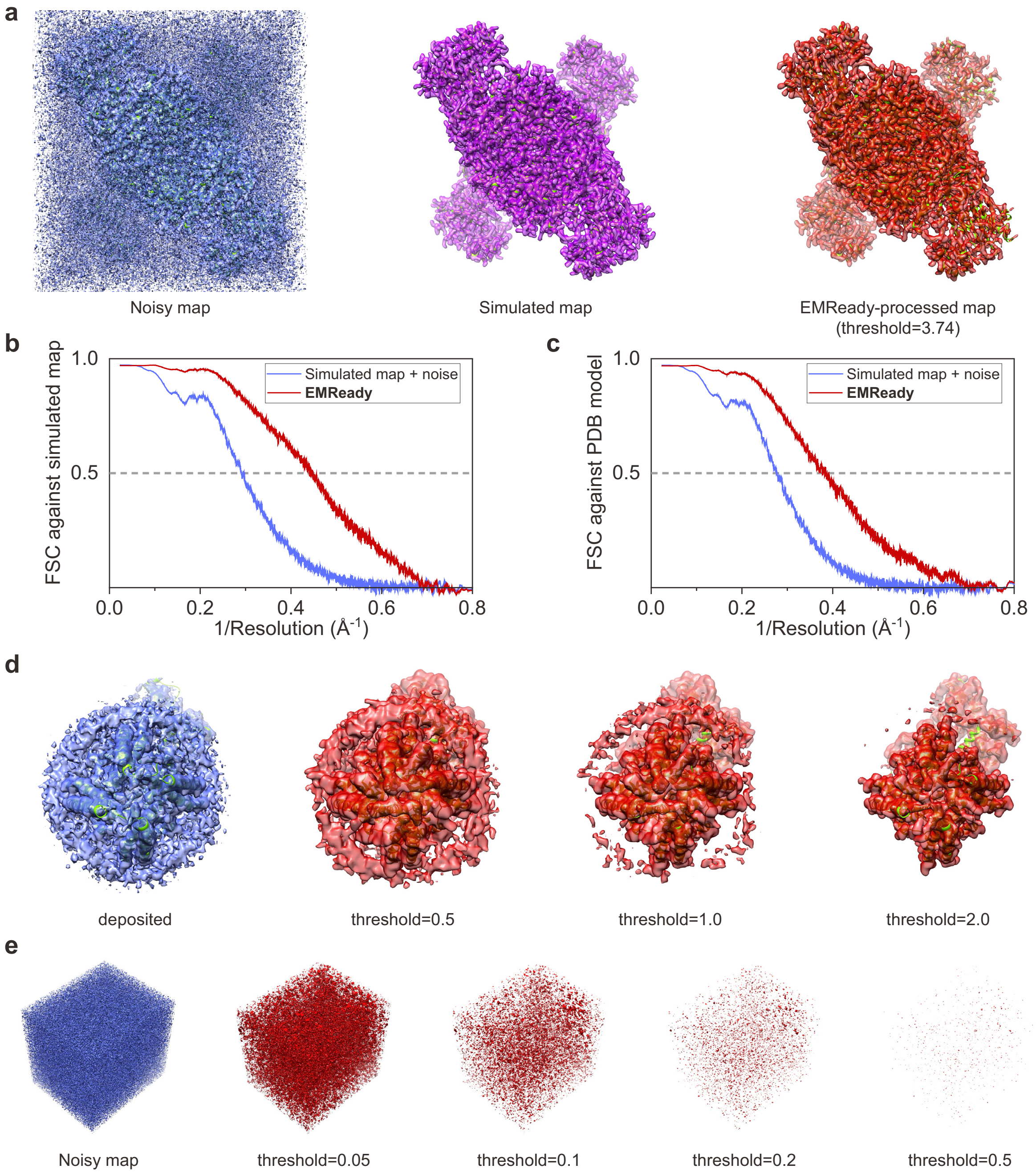

Supplementary Fig. 6: Analysis of the impact of noise on EMReady. a–c Application of EMReady on a simulated map (PDB ID: 6O0H) with very high level of gaussian noise. The density volumes of the simulated map with noise (i.e. noisy map) (blue, *left panel*), the simulated map (purple, *middle panel*), and the noisy map processed by EMReady (red, *right panel*) are displayed in (**a**). The density of the simulated map is normalized to 1.0. Gaussian noise with a standard deviation of 1/6 is added to the simulated map to create the noisy map. Contours are set to 0.25 for both the simulated map and the noisy map. The contour of the EMReady-processed map is drawn to enclose equal volumes to that of the simulated map. The structure of 6O0H is colored in green. The unmasked Fourier Shell Correlation (FSC) curves against the simulated map (**b**) and the PDB model (**c**) are compared between the noisy map and the EMReady-processed map. **d** Comparison of the noises in the deposited primary map (blue) and the EMReady-processed maps at different low contour thresholds (red) for EMD-22216. The associated PDB structure (PDB ID: 6XJX) is colored in green. **e** Application of EMReady on a map of pure Gaussian noise. The noisy map is colored in blue. The EMReady-processed map is colored in red and shown at different contour thresholds. Source data are provided as a Source Data file.

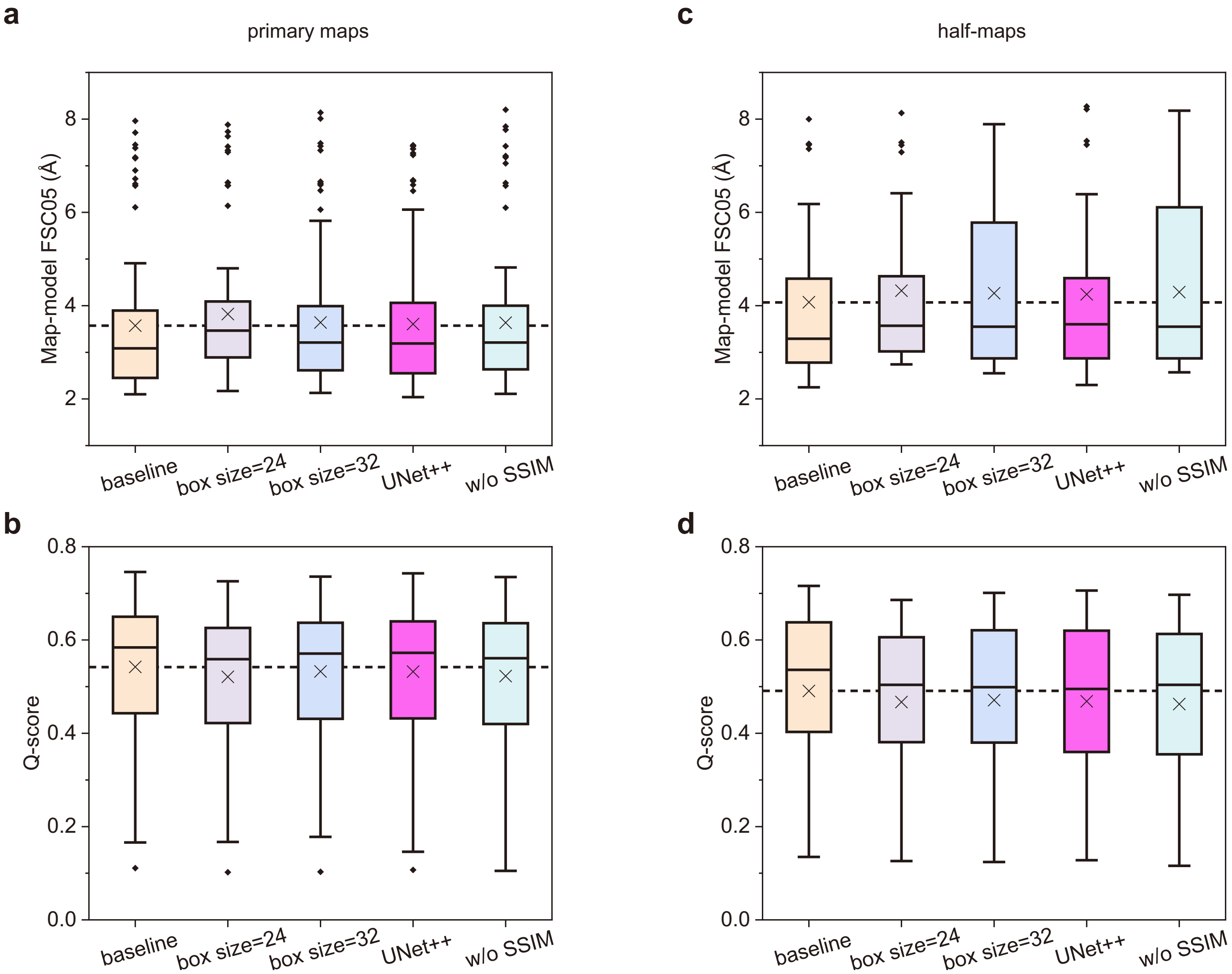

Supplementary Fig. 7: Comparison between the baseline EMReady model and the ablation models in terms of unmasked map-model FSC-0.5 and Q-score. a, b Box-whisker plots of the unmasked map-model FSC-0.5 (**a**) and Q-score (**b**) values on the test set of n=110 deposited primary maps. **c, d** Box-whisker plots of the unmasked map-model FSC-0.5 (**c**) and Q-score (**d**) values on the test set of n=25 pairs of half-maps. The center line is the median, the cross is the mean, lower and upper hinges represent the first and third quartile, the whiskers stretch to 1.5 times the interquartile range from the corresponding hinge, and the outliers are plotted as diamonds. The dashed lines stand for the average values of the baseline model. Source data are provided as a Source Data file.

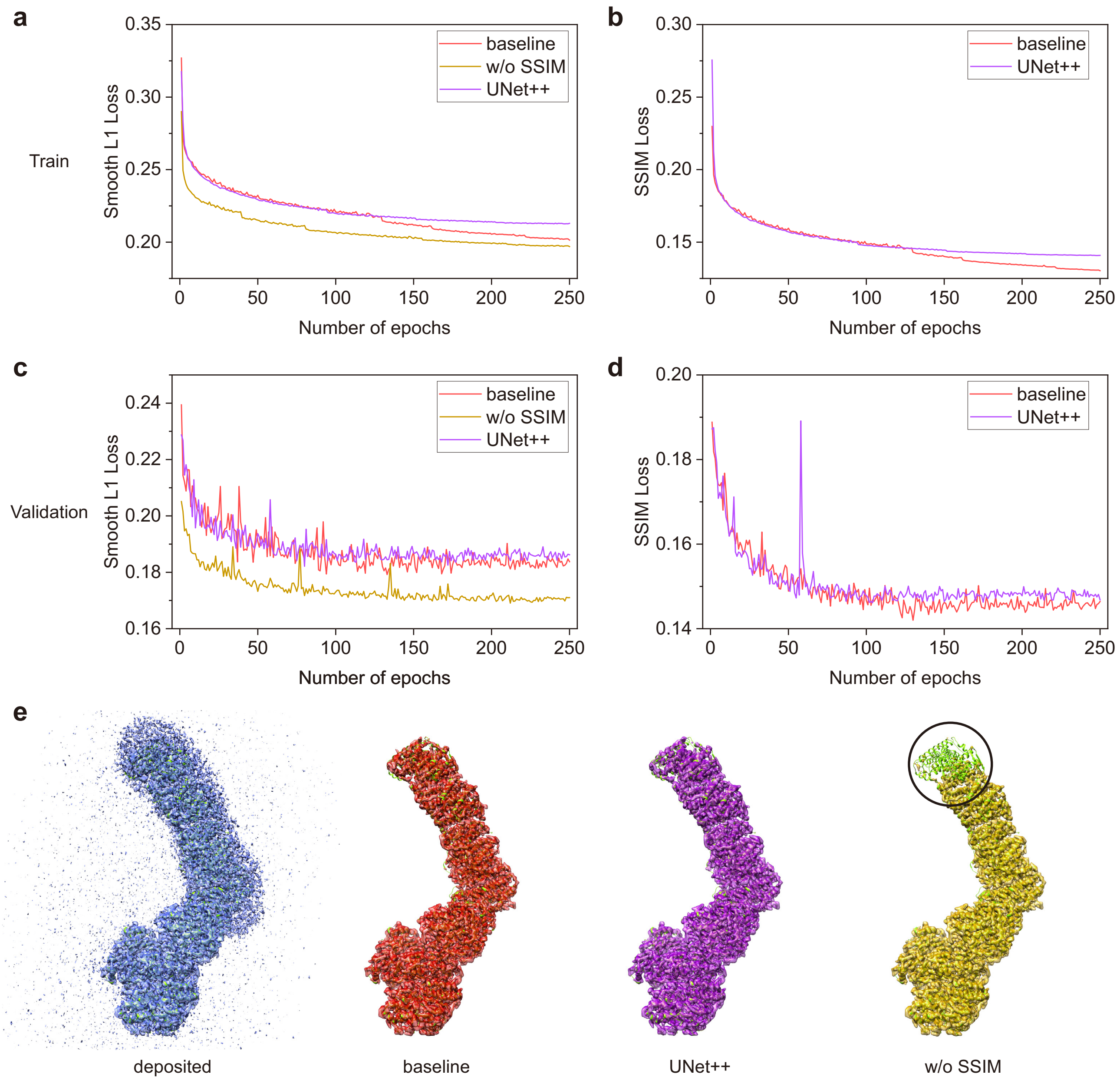

Supplementary Fig. 8: Ablation experiments of EMReady. **a, c** The learning curves of Smooth L1 loss for the EMReady baseline model, the EMReady ablation model with UNet++ architecture, and the EMReady ablation model trained without SSIM loss in the training stage (**a**) and validation stage (**c**), respectively. **b, d** The learning curves of SSIM loss for the EMReady baseline model and the EMReady ablation model with UNet++ architecture in the training stage (**b**) and validation stage (**d**), respectively. **e** Comparison of the density volumes for the deposited primary map of EMD-11231, the map processed by the EMReady baseline model, and the maps processed by ablation models. Contours are drawn to enclose equal volumes. Highlighted in the circle is the density region of a part of the Nqo12 subunit (PDB ID: 6ZIY) that is mistakenly filtered out by the EMReady ablation model trained without SSIM loss. Source data are provided as a Source Data file.

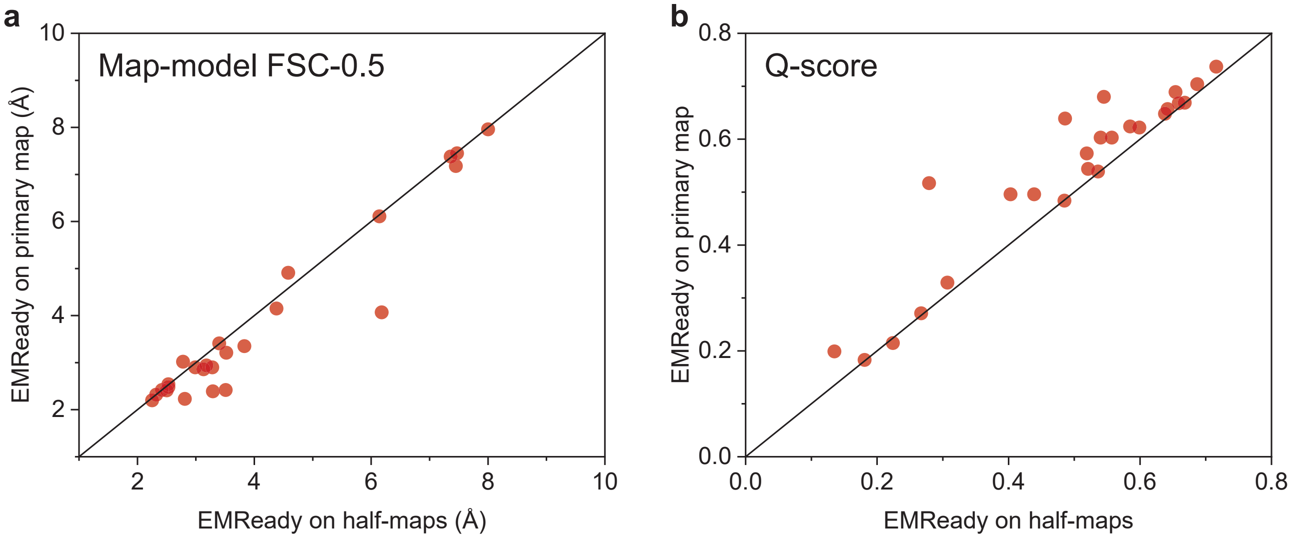

Supplementary Fig. 9: Comparing the evaluation results between using unprocessed half-maps and using post-processed primary map as the input of EMReady on the test set of 25 pairs of half-maps. a Unmasked map-model FSC-0.5. **b** Q-score. Source data are provided as a Source Data file.

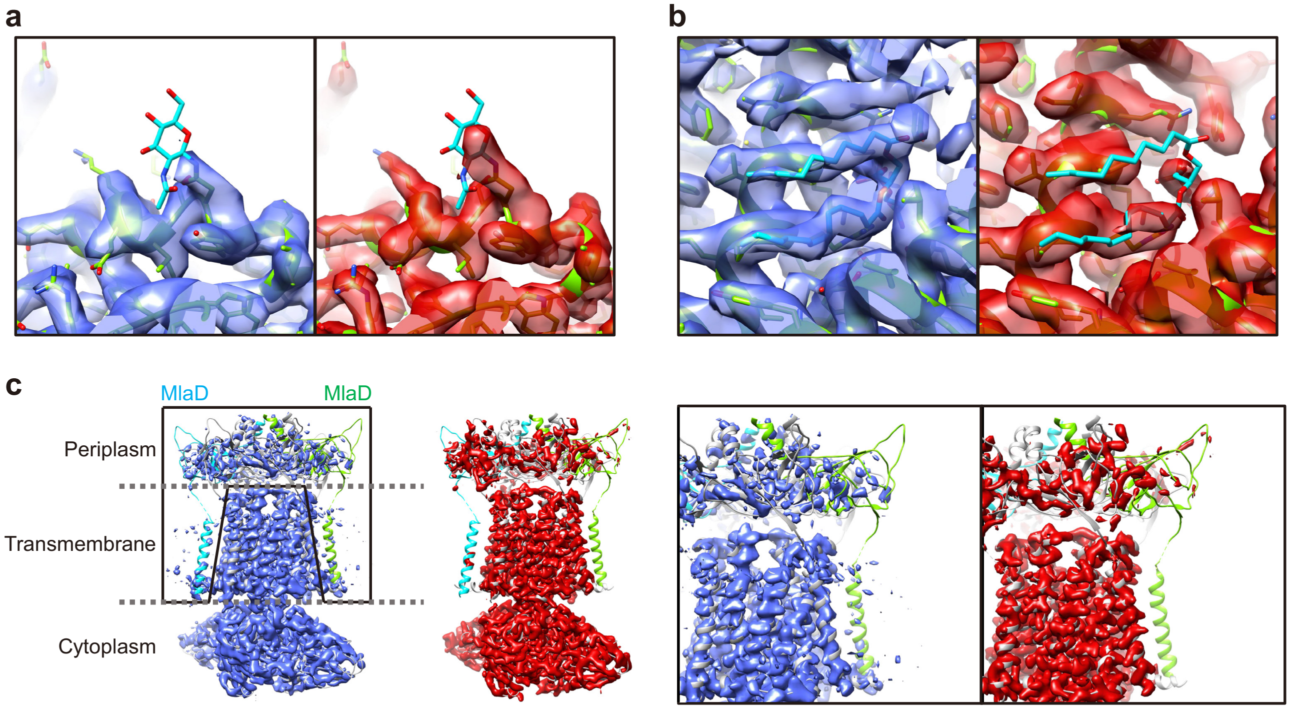

Supplementary Fig. 10: Possible mis-interpretations by EMReady. a Comparison of the density volumes around an N-acetyl-D-glucosamine (Chemical ID: NAG) glycosylated asparagine between the deposited primary map (blue) and the EMReady-processed map (red) for EMD-22112 at 3.5 Å resolution. The density signal of the glycan (cyan) is slightly enhanced by EMReady. **b** Comparison of the density volumes around a phosphatidyl serine (Chemical ID: P5S) between the deposited primary map (blue) and the EMReady-processed map (red) for EMD-0872 (blue) at 3.48 Å resolution. The density signal of the lipid molecule (cyan) is suppressed by EMReady. **c** Comparison between the deposited primary map (blue) and the EMReady-processed map (red) for EMD-30358 (4.3 Å resolution). The density signals of two MlaD subunits (cyan and green) are suppressed by EMReady due to their extremely low quality.

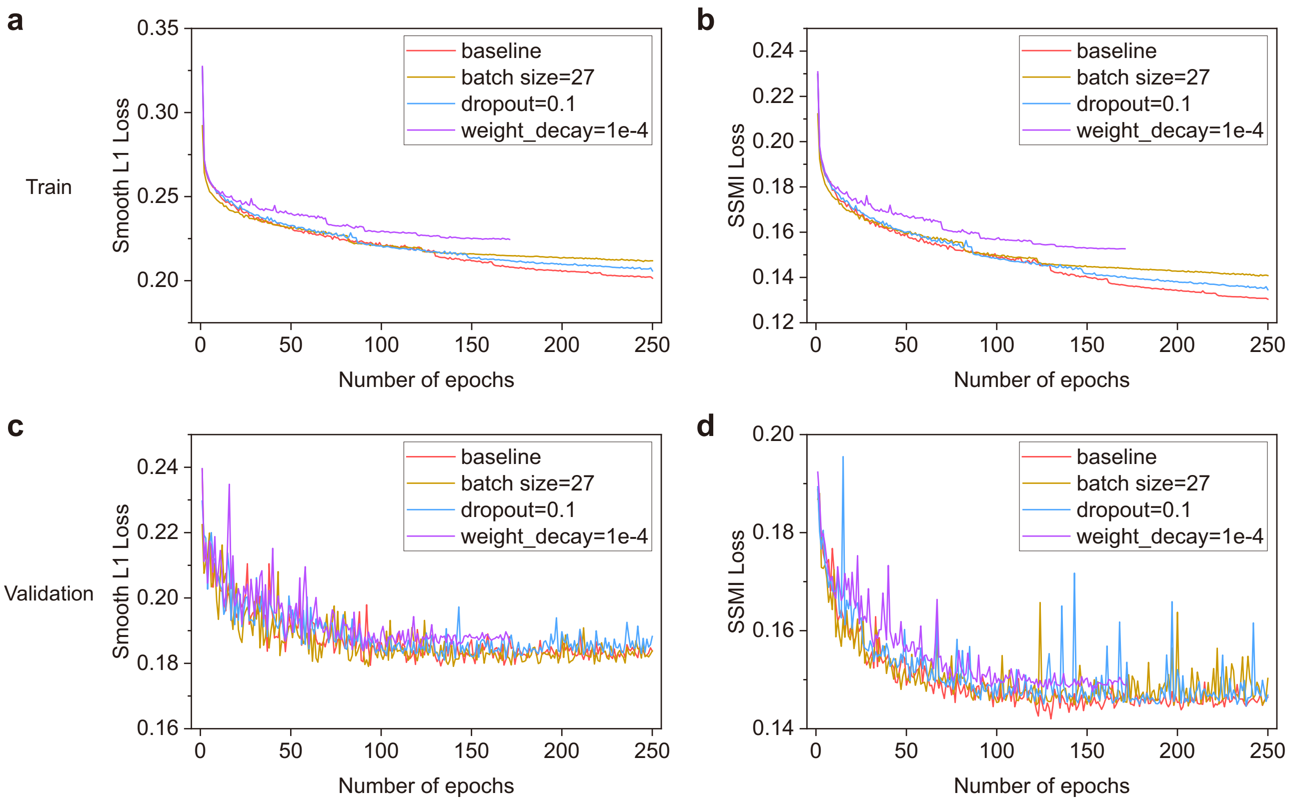

Supplementary Fig. 11: Learning curves for EMReady with different hyperparameters. a, b Comparison of the learning curves for Smooth L1 loss (**a**) and SSIM loss (**b**) in the training stage for the baseline model and the models using a batch size of 27, a dropout rate of 0.1, and a weight decay of 1e-4, respectively. **c, d** Comparison of the learning curves for Smooth L1 loss (**c**) and SSIM loss (**d**) in the validation stage for the baseline model and the models using a batch size of 27, a dropout rate of 0.1, and a weight decay of 1e-4, respectively. Source data are provided as a Source Data file.
