# Supplementary material for: Improvement of cryo-EM maps by simultaneous local and non-local deep learning
Source: Nat Commun. 2023 Jun 3;14:3217. doi: 10.1038/s41467-023-39031-1 (PMC10239474; doi:10.1038/s41467-023-39031-1)
Supplement: Supplementary file 3 — Description of Additional Supplementary Files [file 41467_2023_39031_MOESM3_ESM.pdf]

Description of Additional Supplementary Files

File Name: Supplementary Data 1
Description: **Comparing the quality of the deposited and processed maps on the test set of 110 primary maps.**
There are three different DeepEMhancer models ("tightTarget", "wideTarget", and "highRes").
We report the "combinatorial" results of DeepEMhancer, where the results of DeepEMhancer "highRes" models were used on maps with reported resolutions of < 4Å and the results of default "tightTarget" models were used otherwise.
"-" indicates that DeepEMhancer failed to processed this case, or phenix.mtriage fails to give a valid FSC-0.5 value (i.e. the FSC starts below 0.5) for this case.

File Name: Supplementary Data 2
Description: **Comparing the EMReady models with grid sizes of 1.0 Å and 0.5 Å on the test set of 17 primary EM maps with a voxel size < 1.0 Å.**
"-" indicates that phenix.mtriage fails to give a valid FSC-0.5 value (i.e. the FSC starts below 0.5) for this case.

File Name: Supplementary Data 3
Description: **Comparing the quality of the deposited and processed maps on the test set of 25 pairs of half-maps.**
There are three different DeepEMhancer models ("tightTarget", "wideTarget", and "highRes").
We report the "combinatorial" results of DeepEMhancer, where the results of DeepEMhancer "highRes" models were used on maps with reported resolutions of < 4Å and the results of default "tightTarget" models were used otherwise.
DeepEMhancer failed to process some maps, of which the correponding cases are marked by "-".

File Name: Supplementary Data 4
Description: **Comparion of the FSC-0.5 between the unprocessed half-maps and the FSC-0.5 between the EMReady-processed half-map and the unprocessed half-map on the test set of 25 pairs of half-maps.**

File Name: Supplementary Data 5
Description: **Comparing the models built by phenix.map_to_model for the deposited and processed maps on the test set of 682 chains.**
There are three different DeepEMhancer models ("tightTarget", "wideTarget", and "highRes").
We report the "combinatorial" results of DeepEMhancer, where the results of DeepEMhancer "highRes" models were used on maps with reported resolutions of < 4Å and the results of default "tightTarget" models were used otherwise.
DeepEMhancer failed to process some maps, of which the correponding cases are marked by "-".

File Name: Supplementary Data 6
Description: **Comparing the models built by MAINMAST for the deposited and processed maps on the test set of 385 protein chains.**
There are three different DeepEMhancer models ("tightTarget", "wideTarget", and "highRes").
We report the "combinatorial" results of DeepEMhancer, where the results of DeepEMhancer "highRes" models were used on maps with reported resolutions of < 4Å and the results of default "tightTarget" models were used otherwise.

DeepEMhancer failed to process some maps, of which the correponding cases are marked by "-".

File Name: Supplementary Data 7
Description: **Comparing the EMReady baseline model with the ablation models on the test set of 110 primary maps.**
"-" indicates that phenix.mtriage fails to give a valid FSC-0.5 value (i.e. the FSC starts below 0.5) for this case.
The p-value of each ablation model relative to the baseline model is determined by the Wilcoxon signed-rank test.

File Name: Supplementary Data 8
Description: **Comparing the EMReady baseline model with the ablation models on the test set of 25 pairs of half-maps.**
The p-value of each ablation model relative to the baseline model is determined by the Wilcoxon signed-rank test.

File Name: Supplementary Data 9
Description: **Training and validation losses of the EMReady baseline model and ablation models.**

File Name: Supplementary Data 10
Description: **The network architecture of EMReady.**

File Name: Supplementary Data 11
Description: **The training and validation cases of EMReady.**

File Name: Supplementary Data 12
Description: **Comparing the EMReady models trained with different hyperparameters on the test set of 110 primary maps.**
"-" indicates that phenix.mtriage fails to give a valid FSC-0.5 value (i.e. the FSC starts below 0.5) for this case.

File Name: Supplementary Data 13
Description: **Comparing the EMReady models trained with different hyperparameters on the test set of 25 pairs of half-maps.**

File Name: Supplementary Data 14
Description: **Training and validation losses of the EMReady models trained with different hyperparameters.**

File Name: Supplementary Data 15
Description: **Links of the EMDB and PDB accession codes used in this study.**
