# Supplementary material for: Improvement of cryo-EM maps by simultaneous local and non-local deep learning
Source: Nat Commun. 2023 Jun 3;14:3217. doi: 10.1038/s41467-023-39031-1 (PMC10239474; doi:10.1038/s41467-023-39031-1)
Supplement: Supplementary file 5 — Reporting Summary [file 41467_2023_39031_MOESM5_ESM.pdf]

nature portfolio

Corresponding author(s): Sheng-You Huang

Last updated by author(s): May 13, 2023

Reporting Summary

Nature Portfolio wishes to improve the reproducibility of the work that we publish. This form provides structure for consistency and transparency in reporting. For further information on Nature Portfolio policies, see our Editorial Policies and the Editorial Policy Checklist.

Statistics

For all statistical analyses, confirm that the following items are present in the figure legend, table legend, main text, or Methods section.

n/a	Confirmed	
☐	☒	The exact sample size (*n*) for each experimental group/condition, given as a discrete number and unit of measurement
☒	☐	A statement on whether measurements were taken from distinct samples or whether the same sample was measured repeatedly
☐	☒	The statistical test(s) used AND whether they are one- or two-sided<br>*Only common tests should be described solely by name; describe more complex techniques in the Methods section.*
☒	☐	A description of all covariates tested
☒	☐	A description of any assumptions or corrections, such as tests of normality and adjustment for multiple comparisons
☐	☒	A full description of the statistical parameters including central tendency (e.g. means) or other basic estimates (e.g. regression coefficient) AND variation (e.g. standard deviation) or associated estimates of uncertainty (e.g. confidence intervals)
☐	☒	For null hypothesis testing, the test statistic (e.g. *F*, *t*, *r*) with confidence intervals, effect sizes, degrees of freedom and *P* value noted<br>*Give P values as exact values whenever suitable.*
☒	☐	For Bayesian analysis, information on the choice of priors and Markov chain Monte Carlo settings
☒	☐	For hierarchical and complex designs, identification of the appropriate level for tests and full reporting of outcomes
☐	☒	Estimates of effect sizes (e.g. Cohen's *d*, Pearson's *r*), indicating how they were calculated

Our web collection on statistics for biologists contains articles on many of the points above.

Software and code

Policy information about availability of computer code

Data collection	Greedy algorithm for dataset clustering (written in-house).
Data analysis	Chimera (version 1.15, https://www.cgl.ucsf.edu/chimera/) for data visualization;<br>DeepEMhancer (version 0.14, https://github.com/rsanchezgarc/deepEMhancer);<br>EMReady (version 1.0, http://huanglab.phys.hust.edu.cn/EMReady/) developed in this study.<br>LocScale (https://git.embl.de/jakobi/LocScale/-/tree/master);<br>LocSpiral (https://github.com/1aviervargas/LocSpiral-LocBSharpen-LocBFactor-LocOccupancy);<br>MAINMAST (https://kiharalab.org/emsuites/mainmast.php);<br>MapQ (version 1.6.4, https://github.com/gregdp/mapq) for Q-score calculation;<br>Phenix (version 1.19.2-4158, https://phenix-online.org/);<br>Python (version 3.8.8, https://www.python.org/);<br>PyTorch (version 1.8.1+cu111, https://pytorch.org/) for deep learning framework implementation;<br>Scipion (version v3.0.10, http://scipion.i2pc.es/) for using MonoRes and Xmipp3.

For manuscripts utilizing custom algorithms or software that are central to the research but not yet described in published literature, software must be made available to editors and reviewers. We strongly encourage code deposition in a community repository (e.g. GitHub). See the Nature Portfolio guidelines for submitting code & software for further information.

Data

Policy information about availability of data

All manuscripts must include a data availability statement. This statement should provide the following information, where applicable:

- Accession codes, unique identifiers, or web links for publicly available datasets
- A description of any restrictions on data availability
- For clinical datasets or third party data, please ensure that the statement adheres to our policy

Data that support the findings of this study are available at http://huanglab.phys.hust.edu.cn/EMReady/data/ or from the corresponding author upon request. The Source Data underlying Figs. 2, 3, 4c, d, 5, 6, 7, 8a, c, Tables 1, 2, Supplementary Figs. 2a, b, d, 4a–c, 6b, c, 7, 8a–d, 9, 11, and Supplementary Tables 1, 2 are provided as Source Data file. All published data sets used in this paper were taken from the EMDB and PDB. A full list with links of the EMDB and PDB accession codes used in this study is available in Supplementary Data 15.

Human research participants

Policy information about studies involving human research participants and Sex and Gender in Research.

Reporting on sex and gender	N/A
Population characteristics	N/A
Recruitment	N/A
Ethics oversight	N/A

Note that full information on the approval of the study protocol must also be provided in the manuscript.

Field-specific reporting

Please select the one below that is the best fit for your research. If you are not sure, read the appropriate sections before making your selection.

☒ Life sciences ☐ Behavioural & social sciences ☐ Ecological, evolutionary & environmental sciences

For a reference copy of the document with all sections, see nature.com/documents/nr-reporting-summary-flat.pdf

Life sciences study design

All studies must disclose on these points even when the disclosure is negative.

Sample size

We have collected a non-redundant dataset of cryo-EM maps from the EMDB. All the single-particle EM entries at 3.0–6.0 Å resolutions that have associated PDB models are downloaded from the EMDB and PDB. Any EM map and its corresponding PDB structure that meet the following criteria are removed: (i) containing backbone atoms only, (ii) including unknown residues (UNK), (iii) including missing chain, (iv) having nonorthogonal map axis, and (v) resolution is not given by the FSC-0.143 cut-off. To ensure efficient training, we further exclude those entries with CC mask values less than 0.75. The CC mask values are calculated through the comparison between the deposited EM map and PDB model using phenix.map_model_cc. To remove the redundancy, the remaining cases are clustered using a greedy algorithm. Two models are considered to be similar if any chain in the first model has >30% sequence identity with any chain in the second model. The one with the largest number of similar cases is chosen as the representative of the corresponding cluster, and then the cases in the cluster are removed. This procedure is repeated until all the cases are clustered. The final non-redundant set consists of the representatives of each cluster. A total of 436 pairs of EM maps and associated PDB structures with resolutions ranging from 3.0 Å to 6.0 Å are retained. Out of the total of 436 cases, 86 are randomly selected as the test set, 280 are randomly selected as the training set, and the remaining 70 maps are used as the validation set (Supplementary Data 11).

The initial test set consists of high-quality pairs of maps and PDB models with CC mask values no less than 0.75. We then further collect a supplemental test set of entries with CC mask values between 0.50 and 0.75. Greedy algorithm is also used to remove redundancy in the supplemental set using 30% as the sequence identity cut-off. Moreover, we also exclude the cases in the supplemental set that have >30% sequence identity with any case in the above dataset of 436 cases. After adding 24 cases from the supplemental set, the final test set consists of 110 pairs of maps and structure models, as listed in Supplementary Data 1. As for half-maps, a subset of 25 pairs of half-maps is used, after excluding the cases in the test set that have no corresponding half-maps or have severe mismatch between the map and PDB structure (Supplementary Data 3). For individual chains, the density region within 4.0 Å of each protein or nucleic acid chain is segmented out of the whole primary map. Chains that have mismatch between atomic structure and density volume are excluded. The resulted set consists of 682 pairs of chains and density maps (Supplementary Data 5).

Data exclusions

No data were excluded from the analysis.

Replication

All results could be reproduced by the downloadable package of EMReady, or based on the Methods/Supporting Information.

Randomization	The samples in the non-redundant dataset collected from EMDB and PDB are randomly split into training set, validation set and test set.
Blinding	Blinding is not necessary in this study because the experiments are based on preexisting data from other sources, the analysis is down by software without human intervention and the results are not expected to be influenced by subjective factors.

Reporting for specific materials, systems and methods

We require information from authors about some types of materials, experimental systems and methods used in many studies. Here, indicate whether each material, system or method listed is relevant to your study. If you are not sure if a list item applies to your research, read the appropriate section before selecting a response.

Materials & experimental systems

n/a	Involved in the study
☒	☐ Antibodies
☒	☐ Eukaryotic cell lines
☒	☐ Palaeontology and archaeology
☒	☐ Animals and other organisms
☒	☐ Clinical data
☒	☐ Dual use research of concern

Methods

n/a	Involved in the study
☒	☐ ChIP-seq
☒	☐ Flow cytometry
☒	☐ MRI-based neuroimaging
